# Influence of Pre-Pressing Ring on the Weld Quality of Ultrasonically Welded Short Carbon Fiber Reinforced Nylon 6 Composite

**DOI:** 10.3390/polym14153115

**Published:** 2022-07-30

**Authors:** Zengguo Tian, Qian Zhi, Guopeng Zhang, Xinrong Tan, Lei Lu, Peichung Wang, Zhongxia Liu

**Affiliations:** 1 School of Physics and Microelectronics, Zhengzhou University, Zhengzhou 450001, China; tianzg@zzu.edu.cn (Z.T.); gpzhang@zzu.edu.cn (G.Z.); 2 School of Materials Science and Engineering, Hunan University of Science and Technology, Xiangtan 411201, China; tanxinrong0@163.com; 3 Haima Automobile Co., Ltd., Zhengzhou 450001, China; luleizzu@163.com; 4 Manufacturing Systems Research Lab, General Motors Global Research and Development Center, 30500 Mound Road, Warren, MI 48090, USA; pei-chung.wang@gm.com

**Keywords:** ultrasonic welding, C_f_/Nylon 6 composite, pre-pressing ring clamp, joint strength

## Abstract

The ultrasonic welding (UW) technique is a fast-joining process; it is very suitable for the carbon fiber reinforced thermoplastic (CFRTP) composite. For improving the consistency of the welded joint quality, a new pre-pressing ring clamp (PPRC) was designed for ultrasonic welding carbon fiber reinforced nylon composites in this paper. The effects of the PPRC on the weld quality of the ultrasonic welding welded 4.0 mm thick 30% mass short carbon fiber reinforced Nylon 6 composite was investigated and compared with that of normal clamp weld joint. The weld strength, microstructure, and temperature evolution of the joint were analyzed by tensile test, scanning electron microscope, and temperature measurement. The results showed that the PPCR UW joints had larger central weld nugget size (478 mm^2^ vs. 300 mm^2^), thicker stable fusion region thickness (1.10 mm vs. 0.96 mm), resulting in a higher joint strength (6.86 kN vs. 6.21 kN) compared with the normal clamp UW joints under the same welding parameters. The real-time monitor curve of the horn displacement and temperature at the faying interface showed that the PPRC increased the heat rating at the faying interface during instable melting stage. The PPRC could improve the contact condition between workpieces and the utilization efficiency of ultrasonic energy, which boosted the melting rate of materials at faying interface and consequently the formation of a sound joint with enough weld size (i.e., 433 mm^2^) in a shorter welding time (i.e., 1.3 s). Therefore, the flexibility of component assembly would be increased by the use of this sort of clamps.

## 1. Introduction

Air pollution resulting from automobile exhaust emission has become more and more severe recently. To address this issue, automobile lightening has been proposed as an alternative solution [1,2]. Automobile lightening is using lightweight materials, such as aluminum alloy, magnesium alloy, and polymeric composites, to substitute traditional automobile materials [3,4,5]. Among all the lightweight materials available, carbon fiber reinforced Nylon 6 (C_f_/Nylon 6) composite is a competitive candidate due to its excellent combination of low density and mechanical properties [6,7,8,9,10]. In the application of carbon fiber reinforced Nylon 6 composite to the automotive industry, joining was inevitable. There are many ways for joining C_f_/Nylon 6 composite, such as mechanical fastening, adhesive bonding, and welding. Ultrasonic welding turns out to be an effective way because it is fast, energy efficient, suitable for mass production, and offers good cosmetic quality [11,12,13,14,15].

The quality of an ultrasonic weld can be affected by many influential factors, for instance, material property, weld parameters, contact condition between workpieces, and so forth [16,17,18]. Contact condition becomes a determining factor when the material and welding parameters are optimal. Literatur showed that the contact between upper and lower workpieces had a significant effect on the weld quality of the joint [19,20]. Bates et al. [16] studied the effect of imperfect mating parts on the vibration welding of polyamide 6 compound and found the meltdown profile and part quality were affected greatly by the imperfect contact between the welded parts. Based on our previous studies [21,22,23], the imperfect contacts of the horn and workpiece or upper and lower workpieces influenced the formation process of the weld area, which was closely related to the weld quality of the joints (e.g., squeezed melt flow, splash, severe horn indentation, etc.). Zhi [23] tried to keep the upper workpiece flat to improve the contact between the horn and workpiece, as a result, the joint strength and the cosmetic quality improved simultaneously.

The imperfect contact can be improved by increasing the weld pressure, but excessive pressure would bring issues such as splash, squeezed oute melt, and decreased joint strength. Wijk [24] investigated the effect of weld pressure on the weld quality of ultrasonic welded ABS polymers and find the optimal weld pressure was in the range of 200–300 N while the joint strength decreased significantly with further increasing weld pressure. Matsuoka [25] welded glass fiber reinforced plastics by ultrasonic and found that the increase of weld pressure would increase the joint strength as well as shorten the optimal weld time. Zhang [26] studied the influence of weld pressure on the joint strength of ultrasonic welded PLA and PMMA, the maximum strength occurred when the weld pressure approached 0.4 MPa, and the strength dropped once the weld pressure exceeded 0.4 MPa. Yang [27] investigated the effect of weld pressure on the contact condition during ultrasonically welded polymers. It was found the contact between workpieces was not intimate and the effective friction cannot be obtained when the weld pressure is lower and thus the energy efficiency and weld quality are unsatisfied. However, the molten flash moved quickly and squeezed out when the weld pressure is too higher, resulting in a decrease in joint strength due to the deficiency of molten materials. Besides, excessive weld pressure would bring a great load to the welder and make the welding process difficult.

**Figure 1:**
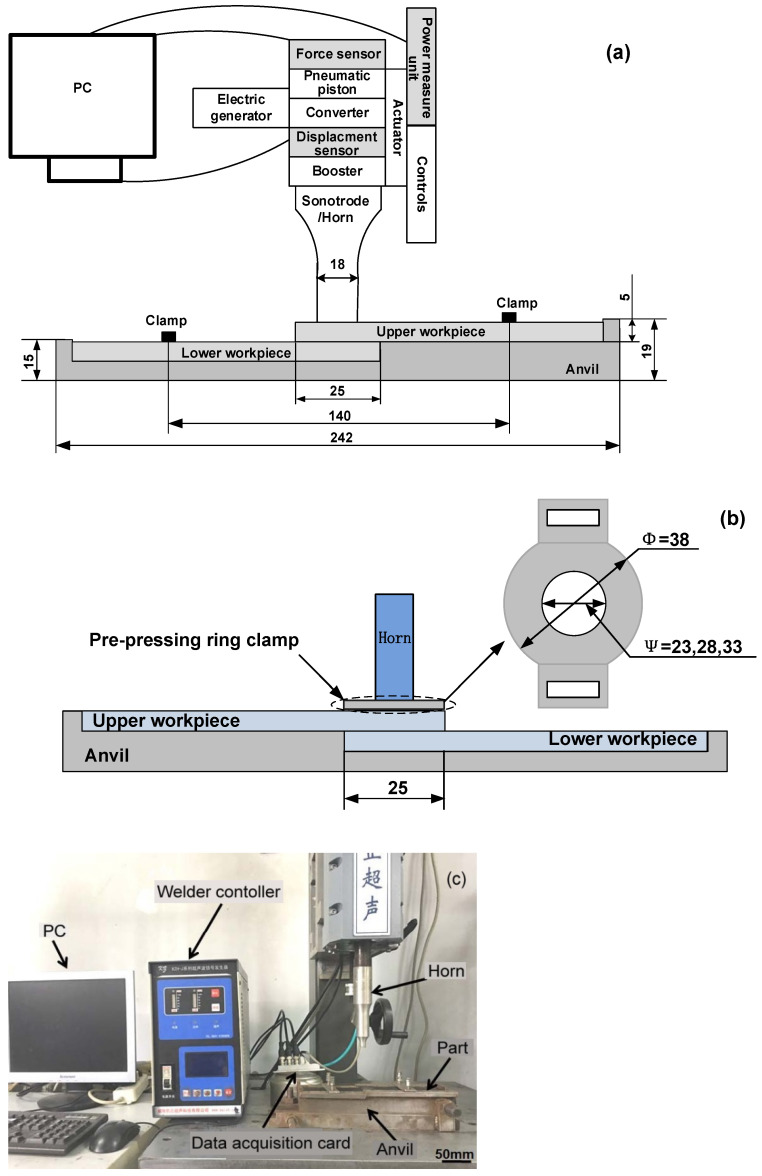
Schematic of ultrasonic welding of injection molded 4 mm thick lapped Cf/PA6 composite without energy director: (**a**) normal UW; (**b**) with PPRC; (**c**) Ultrasonic welder (Dimensions in mm).

Though it has been demonstrated that the loose contact was detrimental to the weld quality, little work has been conducted to improve the joint performance. In mass assembly, in order to ensure close contact between two welding parts and improve the consistency of the welded joint quality, a pre-pressing clamp around the weld area was proposed in this study. The normal clamp and designed PPRC are shown in Figure 1a,b. The PPRC was placed and fixed at the periphery of the weld area and the horn was indented into the designed circle with different radii, which would provide higher pressure to the workpieces during the welding process than that of the normal clamp. To distinguish it from normal ultrasonic welding (NUW), it was defined as PPRC fixture ultrasonic welding (PPRCUW).

In this study, PPRC with various diameters was designed and applied to UWed C_f_/Nylon 6 joint to improve the contact between upper and lower workpieces. The effect of different clamps on the strength and weld area of the joint were investigated firstly. Then, the transient horn displacements and temperature evolutions at the faying interface for the ultrasonic welds with different clamps were recorded and compared. Microstructures and the failure modes of these joints were also evaluated. Finally, the influential mechanism of PPRC on the weld joint was discussed. This study provides a new approach and good understanding of the effect of PPRC on the characteristics of UWed C_f_/Nylon 6 composite.

## 2. Experimental Procedure

### 2.1. Materials

Commercial polyamide 6 (Polibend Engineering Plastics, Italy) with a purity of 99% and carbon fiber (24K, T300 type, Toray Carbon Magic Co., Ltd., Maibara-shi, Japan) with a length of 2 mm were used. The fibers were first cleaned with a concentrated solution of nitric acid and then surface pretreated using 8% diglycidyl ether of bisphenol solution in acetone. Both polyamide 6 and pretreated carbon fibers were dried at 80 °C in a vacuum condition for 3 h before being used to fabricate 30 wt.% carbon fiber/polyamide 6 composite.

A twin-screw extruder with two separate inlets was used to mold 30 wt.% carbon fiber/polyamide 6 composite. Polyamide 6 was added to the first hopper, and carbon fibers were added to the second hopper. Polyamide 6 was fully melted before carbon fibers were added to minimize the fracture of carbon fiber during compounding. The processing temperature was 270–280 °C, and the screw speed was 180 rpm. After fully mixing polyamide 6 with carbon fibers in a twin-screw extruder, carbon fiber/polyamide 6 composite were processed into the pellets with a length of 2 mm. The pellet was then fed into the injection extruder to mold into coupons with dimensions of 132.0 × 38.0 × 4.0 mm. All coupons were stored in an ambient laboratory environment (i.e., 20 °C and 50% R.H.) and dried in a vacuum oven at 80 °C for 48 h before welding to completely remove moisture in the specimen. The mechanical properties of the molded composite are shown in Table 1.

### 2.2. Ultrasonic Welding Process

Ultrasonic welding process was performed using a KZH-2026 multi-function UW machine (Weihai Kaizheng Ultrasonic Technologies Co., Ltd., Weihai, China) with a nominal power of 2.6 kW, nominal frequency of 20 kHz, and nominal amplitude of 25 μm. The machine (Figure 1c) was equipped with a data acquisition system that combined with a horn pressure sensor, horn-displacement sensor, and timer, which were integrated into the controller of the UW machine, as shown in Figure 1a. In addition, the horn pressure, weld energy, and displacement of the horn were recorded online on a PC as a function of time by the data acquisition system. The final horn displacement, weld energy, weld time, horn pressure, hold time, and delay time were also displayed in the control panel during the UW process. To avoid coupon motion during welding, the coupons were held in place by using a fixture.

The machine had three welding modes, namely, energy, time, and collapse modes. The value of weld energy, weld time, and horn displacement for the three modes, respectively, were preset to control the welding process. The workpieces were then welded using the nominal power of the machine. When the weld energy, weld time, or horn-displacement reached the preset values for the selected weld mode, the ultrasonic wave oscillation was stopped. Therefore, weld quality was controlled by the preset values in each selected welding mode. When time mode was selected, the values of the delay time, weld force, hold time, and ultrasonic time were preset prior to welding. When the ultrasonic triggering was performed, the horn was pressed onto the workpieces for two seconds, and then the ultrasonically vibrated until the preset time was reached. The welded workpieces were held for three seconds to solidify the molten material. All specimens were welded using a 7075 aluminum horn with a diameter of 18 mm.

### 2.3. Temperature Measurement

**Figure 2:**
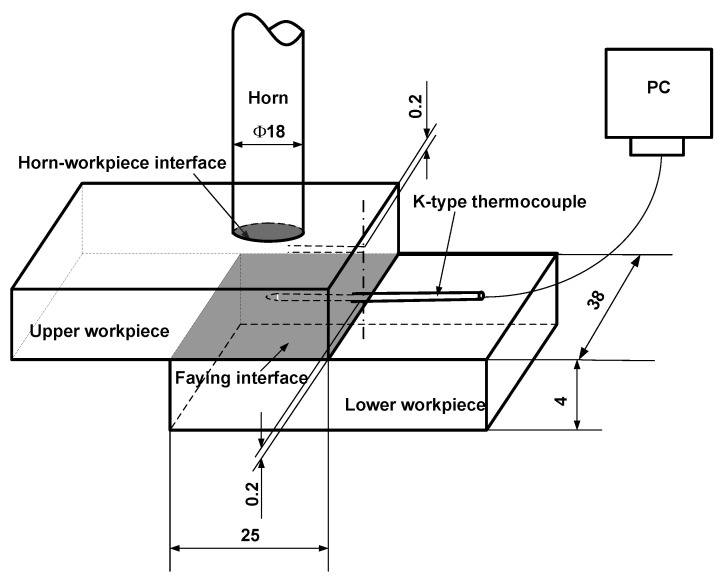
Sketch of the temperature measurement during the ultrasonic welding of injection molded 4 mm thick lapped C_f_/PA 6 composite without energy director (Dimensions in mm).

In order to analyze the weld initiation and growth during the ultrasonic welding, the temperature evolution at the location near the faying surface was measured. Figure 2 shows the experimental setup for temperature measurement. As shown, a small hole with a diameter of 1.0 mm and a depth of 12.5 mm was drilled at the side of the upper workpiece. The hole was drilled at 0.2 mm from the bottom surface of the upper workpiece. K-type thermocouple was embedded into the two small holes and secured with epoxy compound so that the thermocouple was secured. The temperature evolution near faying interface was recorded as a function of time by a data acquisition system during the ultrasonic welding.

### 2.4. Weld Microstructure

**Figure 3:**
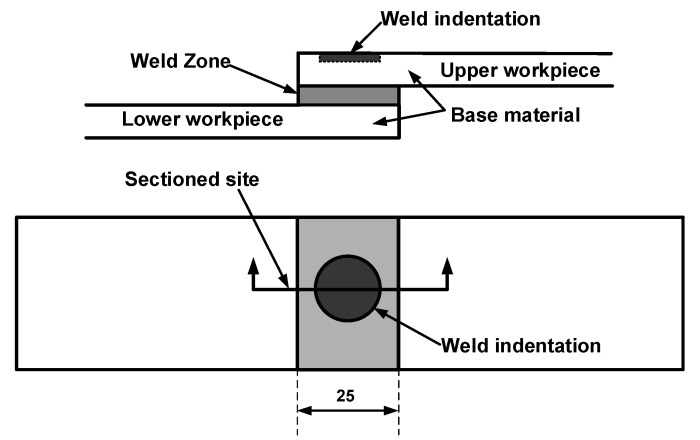
Schematic of sample preparation for examining the microstructure of the ultrasonic welded 4 mm thick injection molded C_f_/PA 6 composite (Dimensions in mm).

To assess the characteristics of the weld microstructure of the ultrasonic welded joints, the specimens were prepared using the procedures shown in Figure 3 for the tested joints. Referring to Figure 3, the joints were notched from the central position of the weld. Then, the pre-notched specimens were immersed in liquid nitrogen for 10 min. The embrittled specimens were broken off from the notched site; the broken specimens were sputter-coated with gold for 50 s to increase the conductivity, and the microstructures of the welds were examined with scanning electron microscope (JSM6700F).

### 2.5. Quasi-Static Test

Quasi-static tests were performed by loading each specimen to failure in an MTS 810 tensile tester per ASTM D1002-2001. To minimize bending stresses inherent in the testing of single-lap weld specimens, filler plates were attached onto both ends of the specimen using a masking tape to accommodate the sample offset. Load vs. displacement results were obtained, as the specimens were loaded at a stroke rate of 2 mm/min. Joint strength is evaluated by peak load in this study. Three replicates were performed, and the average joint loads were reported.

## 3. Result and Discussion

### 3.1. Effect of Dimension of The Pre-Pressing Ring on Joint Strength

**Figure 4:**
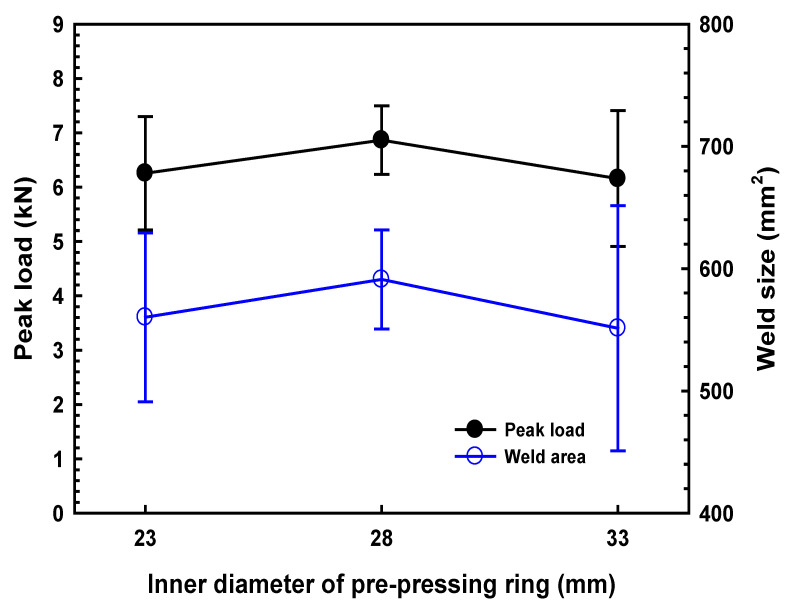
Effect of the diameter of the PPRC on the joint strength and weld size of the UW joints.

To examine the effect of dimension on the joint strength of ultrasonic welded 4 mm thick lap carbon fiber/Nylon 6 composite with 30% weight fiber, three diameters of the PPRC with various internal diameter (i.e., Φ 23, Φ 28 and Φ 33) were selected and fabricated. All UW joints were fabricated under a weld time of 2.1 s and a weld pressure of 0.15 MPa. Figure 4 presents the joint strength and weld size of the UW joints as a function of the diameter of inner ring. Referring to Figure 4, the joint strength and the weld size increased first and then decreased as the increase of the diameter of the PPRC. The welds made with Φ 28 diameter exhibited the highest peak load. Concerning the restrictions of welding position and geometric accuracy of horn position in practice, the effect of the clamp with Φ 28 mm diameter (28 mm clamp) on the weld initiation and formation was investigated primarily.

### 3.2. Effect of The Pre-Pressing Ring on Joint Strength

**Figure 5:**
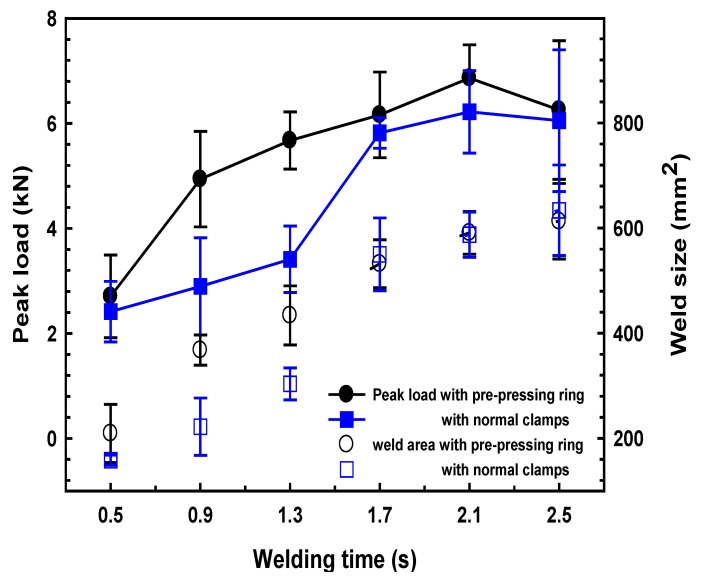
Correlation among the weld time, joint strength, and weld size of the UW joints welded with two kinds of clamps.

Figure 5 presents the effect of welding time on the joint strength and weld size of the UW joints made with the different clamps. Referring to Figure 5, the joint strength of the UW joints showed a similar tendency, which increased firstly with increasing welding time and then decreased. The optimal welding time for the two clamps was 2.1 s and the joint strength reached 6.8 kN and 6.2 kN for UW joints made with PPRC and normal clamp, respectively. The weld size increased with the weld time and then reached a plateau. Careful examination of the results indicated that joint strength and weld size of UW joints made with the PPRC were significantly greater than that of UW joints made with a normal clamp when the welding time increased from 0.9 s to 1.3 s. Hence to obtain a solid joint strength (i.e., >5 kN), the minimum welding time for normal clamp joints was 1.7 s, while it was 1.3 s for joints made with PPRC. The application of the PPRC would likely improve the contacting conditions between upper and lower workpieces, which was beneficial for ultrasonic energy absorption at the faying interface.

**Figure 6:**
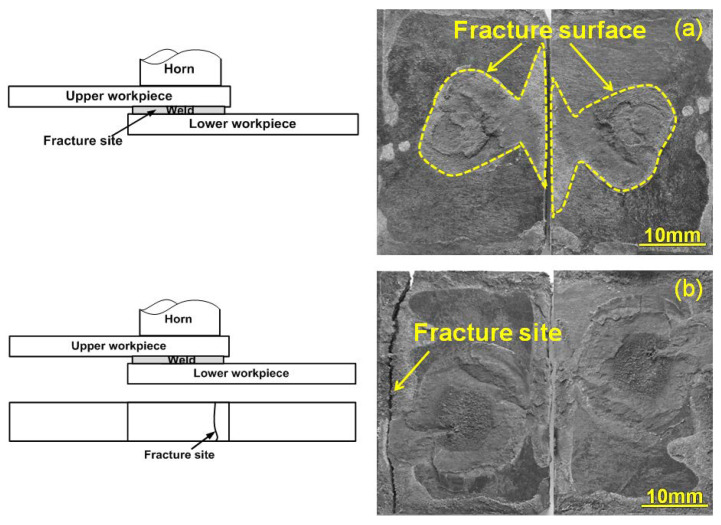
Failure modes for the ultrasonic welded 4 mm thick lap carbon fiber/polyamide 6 composite with 30 wt.% carbon fiber and without energy director: (**a**) interfacial failure; (**b**) mixed interfacial and workpiece fracture.

Visual examinations of the quasi-static tested welds indicated that there were mainly two kinds of fracture modes for the welds regardless of the clamps. Generally, the weld with an insufficient weld area (i.e., welding time of 0.5, 0.9 s for joints made with PPRC; 0.5, 0.9, and 1.3 s for joints made with normal clamp) likely exhibited interfacial failure, referring to Figure 6a. As the weld time prolonged, the weld area at the faying surfaces increased, and consequently led to a workpiece breakage fracture mode shown in Figure 6b. The presence of interfacial failure of joints is mainly because the insufficient weld area cannot bear the tensile force and breaks through the nugget. As the weld area reaches a high level, the fracture is likely to initiate from the edge of the weld area (i.e., the most stress-concentrated region) and show workpiece breakage.

### 3.3. Transient Horn Displacement and Temperature

**Figure 7:**
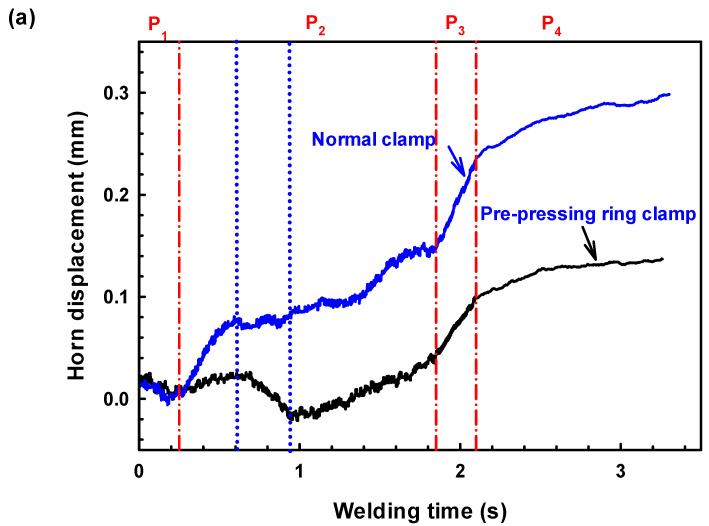

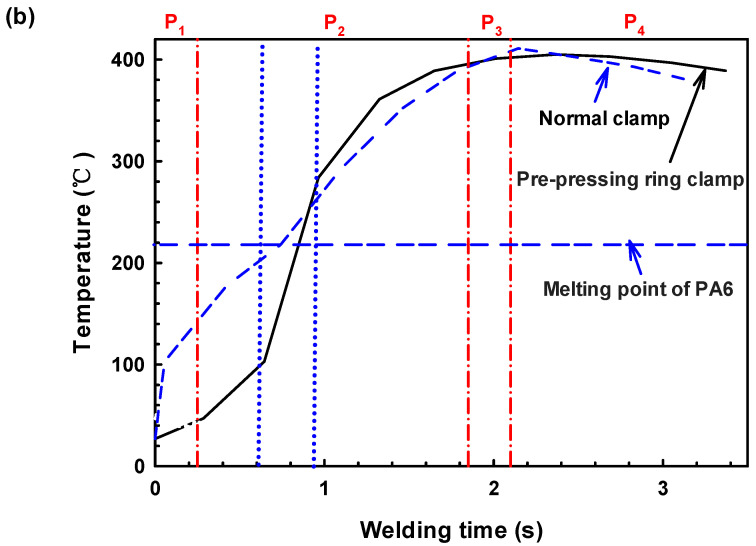
Effect of the pre-pressing ring and normal clamps on the displacement curve of horn (**a**) and weld temperature at the faying interface (**b**).

To analyze the effect of normal and PPRCs on ultrasonic energy absorption during welding, the transient horn displacement and the transient temperature at the faying interface were measured and compared. Figure 7 presents the horn displacement and temperature history as a function of weld time for the UW joints made with the two clamps. Referring to Figure 7, the transient horn displacement for the two joints exhibited similar characteristics. According to literature [22], the two welding processes can be divided into four stages as shown in Figure 7: (1) At the beginning of ultrasonic vibration (i.e., P1, Coulomb friction phase), the micro-scale surface asperities on the workpiece-to-workpiece surfaces heated rapidly and caused volumetric heating. The materials under heating began to expand, which pushed the horn retraction, causing the displacement to decrease. During this stage, the temperatures at the faying interface increased for both normal and PPRCs, while the temperature of normal clamp increased faster due to the loose contact between workpieces. (2) Phase 2 was an unsteady melting phase, volumetric heating continued and temperature increased to above the melting point of the composite during this phase; and the faying interface began to melt and the weld area increased until a stable melt film formed. The temperature for the PPRC increased at a smaller rate. During the late period of Phase 2, the temperature for PPRC is higher than that of the normal clamps during this phase, which is probably because the intimate contact between workpieces prohibited the molten materials to spread out, and the molten materials accumulated at the faying interface. (3) Phase 3 was the steady melting phase, during this phase, the melting rate of the parts and the rate of outflow of the molten material were in equilibrium, which resulted in a constant thickness film. The temperatures increased through hysteresis dissipation and the temperatures increased at a lower rate to the highest point and remained stable for the upper workpiece. (4) At the last phase (P4, solidification phase), ultrasonic vibration stopped, and the materials started to solidify, along with the material contraction under the weld force. Ultimately, the displacement slightly increased and temperatures started to decrease.

It was worth noting that although the UW joints made with the two clamps showed similar P1, P3, and P4 phases, the P2 phase was quite different. Referring to Figure 7, the P1 phase in UW joints made with both two clamps ranged from about 0 to 0.3 s, in which the horn displacement decreased continuously resulting from the expansion of workpieces. Then, the horn displacement non-linearity increased with prolonged oscillation time (i.e., 0.3~1.8 s) for joints made with normal clamp, indicating the beginning of the unstable melting phase (i.e., P2 phase). On the contrary, the horn displacement of UW joints made with a pre-pressing ring clamp firstly increased as the oscillation time reached 0.3~0.6 s caused by the melting of materials in the central nugget area, and then abnormally decreased as the oscillation time increased to 0.6~0.9 s, which likely attributed to the expansion of materials under prepressing ring with vast heating. As oscillation time further increased to 0.9~1.8 s, the horn displacement of joints made with prepressing ring clamp non-linearity increased, which was similar with that of joints made with a normal clamp.

The difference in the P2 phase for UW joints made with the two clamps was likely caused by the different contact conditions in the overlap region. For the UW joints made with a prepressing ring clamp, the overlap region was tightly compressed resulting in difficulty of starting ultrasonic oscillation and even a decrease in the initial amplitude of ultrasonic oscillation. Therefore, the temperature increment at the faying interface for UW joints made with a prepressing ring clamp during the P1 phase was lower than that of UW joints made with a normal clamp (shown in Figure 7b). The prepressing ring clamp effectively improved the contact condition of the overlap area between the upper and lower workpieces and consequently the viscoelastic heat generation. The heating rate of joints made with prepressing ring clamp at the faying interfaces was higher than that of joints made with normal clamp during P2 phase, resulting in a higher interface temperature (even higher than the melting point of PA6) during the latter part of P2 phase (i.e., 0.9~1.8 s), referring to Figure 7b. This phenomenon was mainly attributed to the presence of intimate contacting overlap region was in favor of ultrasonic transmission and absorption during welding which greatly boosted the melting speed at the faying interface.

### 3.4. Weld Nugget Formation and Evolution

**Figure 8:**
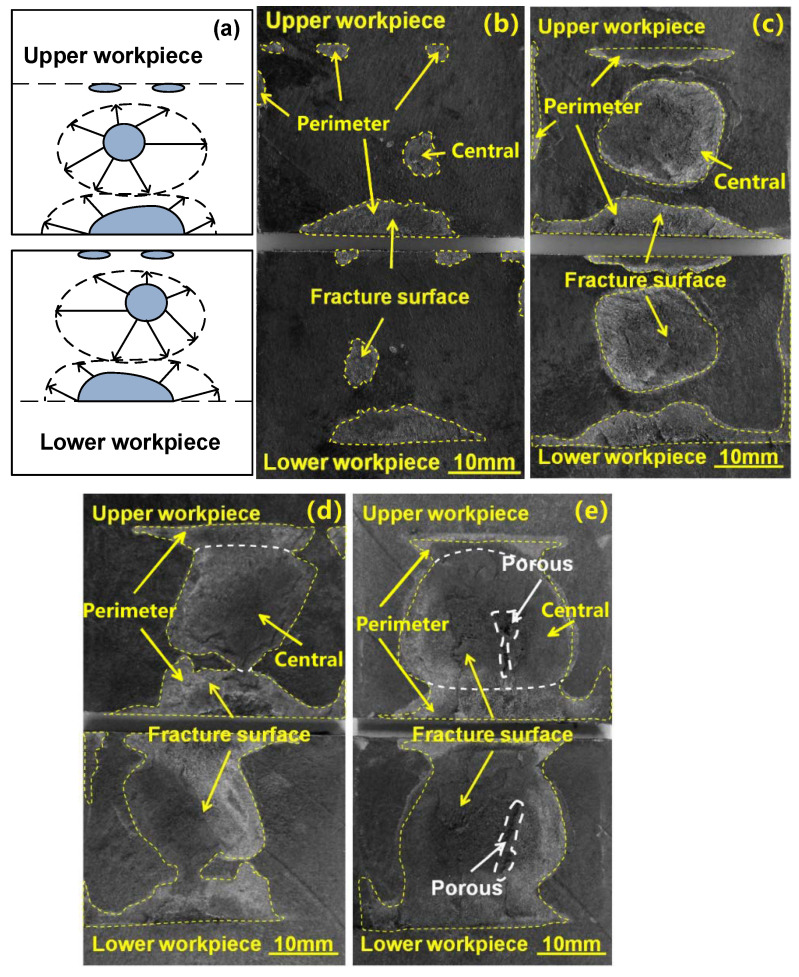
Schematic of weld growth with PPRC (**a**), and weld appearance at various welding times (**b**) 0.5 s, (**c**) 0.9 s, (**d**) 1.3 s, (**e**) 2.1 s.

**Figure 9:**
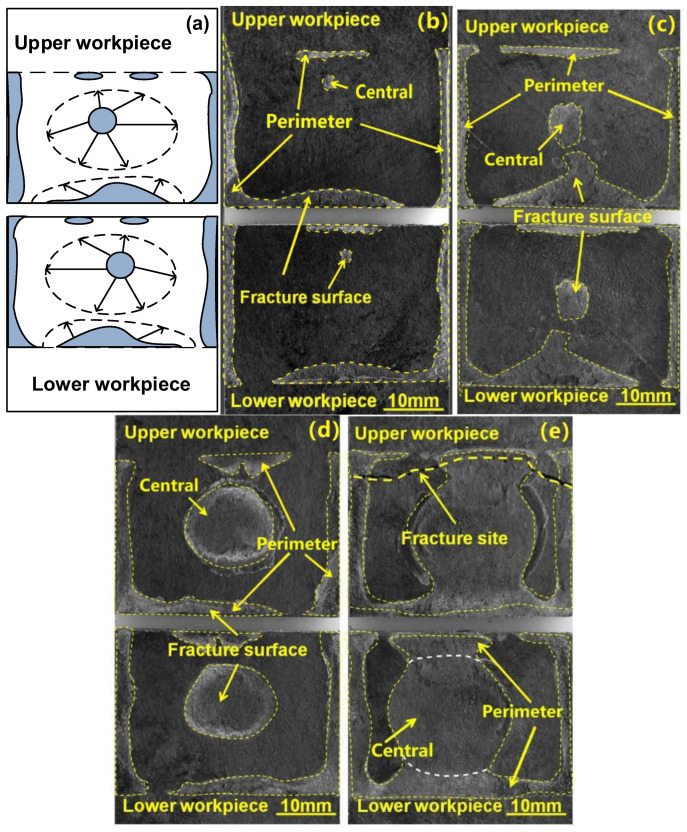
Schematic of weld growth with normal clamps (**a**), and weld appearance at various welding times of (**b**) 0.5 s, (**c**) 0.9 s, (**d**) 1.3 s, (**e**) 2.1 s.

To further understand the effect of PPRC on the weld initiation and growth during the welding of 4 mm thick lap carbon fiber/polyamide 6 composite, the weld growth with various weld times was studied and the results were shown in Figure 8. For comparison, the weld growth of the joint made with a normal clamp is shown in Figure 9. Similar weld formation and evolution phenomenon was observed in joints made with normal and PPRC from the SEM images. Referring to Figure 8 and Figure 9, it was clearly that both the materials at central and the perimeter zone of weld area initially melted resulting in the formation of a central (the middle of the overlap region) and perimeter weld nugget (edges of the overlap), respectively, as welding time was 0.5 s. Then, the central weld nugget grew up rapidly and expanded in all directions with an increase in welding time. Simultaneously, the perimeter weld nugget expanded toward the central of overlap zone. The two weld nuggets finally merged with each other resulting in the formation of a weld since the welding time reached 1.3 and 2.1 s.

**Figure 10:**
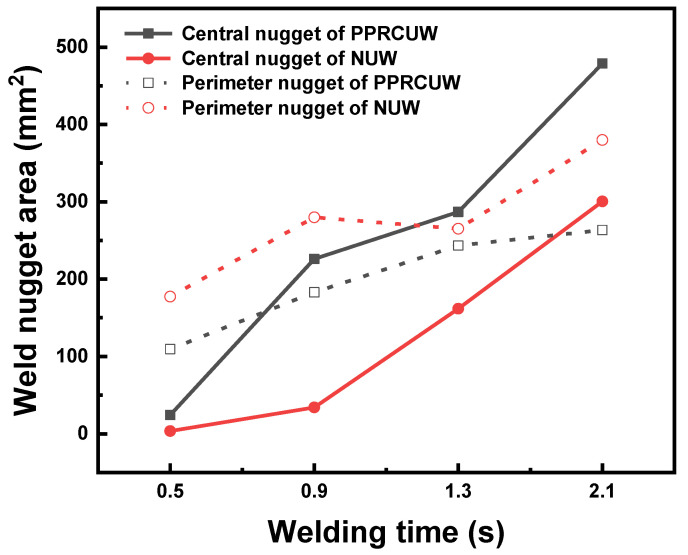
Effects of welding time on weld nugget size of NUW and PPRCUW joints.

Figure 10 shows the weld nugget size variation of NUW and PPRCUW joints during ultrasonic welding. It was worth mentioning that the central weld nugget area for the joint made with PPRC was much greater than that of the normal clamp under the same weld time. This phenomenon was more notable as the welding time was more than 0.5 s. Besides, the grown-up speed of the central weld nugget was faster than that of the perimeter weld nugget, especially for joints made with PPRC. Combination analysis results of Figure 8 and Figure 9 suggested that the PPRC speed up the formation of central weld nugget and weld growth, because the applied force from the PPRC improved the contacting condition of upper and lower workpieces at the center of the overlap region, which was beneficial for the propagation behavior of the ultrasonic wave and enhanced the energy dissipation at the faying interface. Comparing the results in Figure 5 and Figure 10, it can be suggested that the weld strength was mainly determined by the central weld nugget area. This was mainly because a high-stress raiser was developed around the edge of the central weld under an applied loading [12,28]. Therefore, the larger the central weld the higher the joint strength.

Careful examinations of the fractured faying surfaces showed that there existed a small porous region for the weld made with PPRC and 2.1 s weld time (Figure 8e). While the faying surface was relatively compact with a 2.1 s weld time (Figure 9e) and porous region occurred when the weld time was longer than 2.5 s for normal clamp. These characteristics suggested that the temperature in the weld made with 2.1 s was high and resulted in the decomposition of Nylon 6 matrix for PPRC. The results were consistent with the recorded transient temperature history shown in Figure 7. The aforementioned analyses indicated that the PPRC enhanced the energy transmission efficiency of the central of overlap during ultrasonic welding.

### 3.5. Effect of Clamp on Weld Microstructure

**Figure 11:**
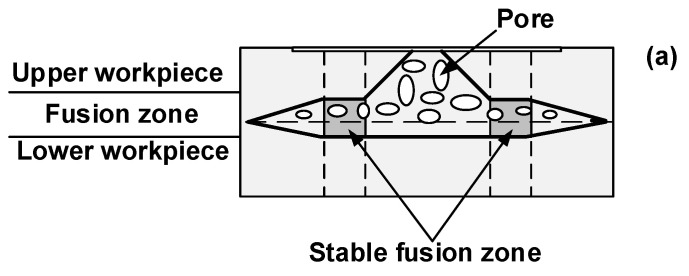

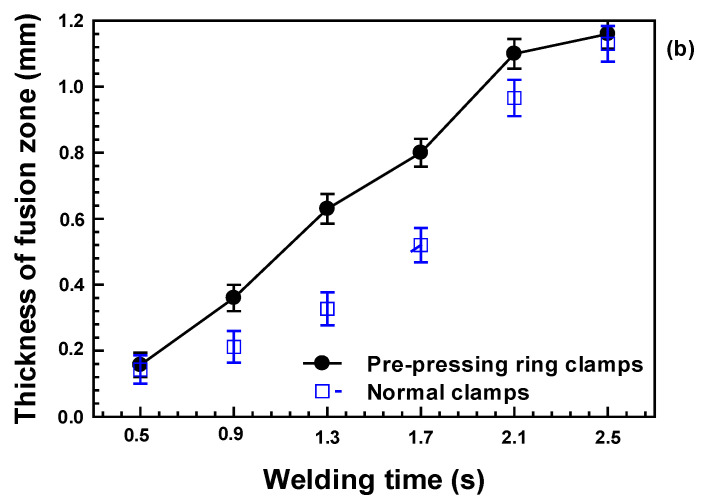
Schematic of stable fusion zone (**a**) and the correlation between the weld time and thickness of fusion zone with two kinds of clamps (**b**).

Literature showed that welding quality was closely correlated with the weld microstructure [21,22]. The thickness of fusion zone can significantly influence the joint strength [29,30]. Hence, the effect of clamp on weld microstructure was investigated. Figure 11a,b show the schematic of stable fusion zone at the weld zone and the correlation between the weld time and thickness of the fusion zone with two kinds of clamps. Referring to Figure 11, the thickness of the fusion zone for UW joints made with the PPRC was greater than that of UW joints made with a normal clamp for the joints made with weld time from 0.5 s to 2.5 s under 0.15 MPa. Those results indicated that the PPRC not only increased the weld size but also the thickness of the fusion zone of UW joints, both of which would be beneficial to improving the joint strength.

**Figure 12:**
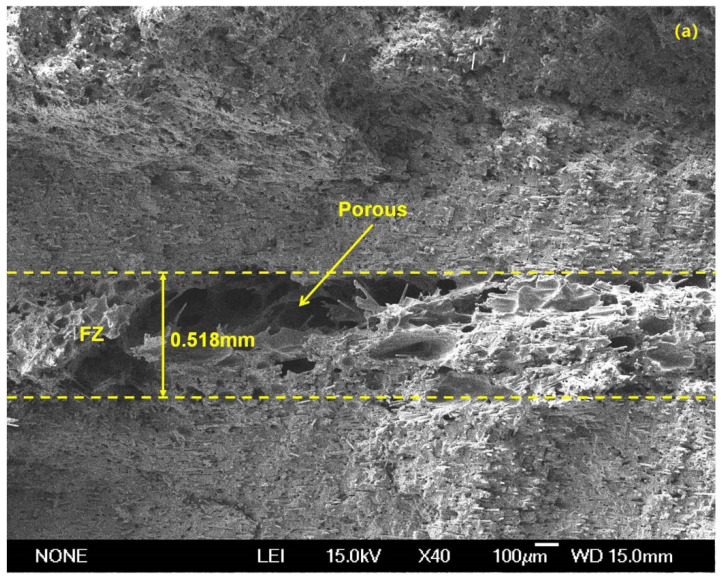

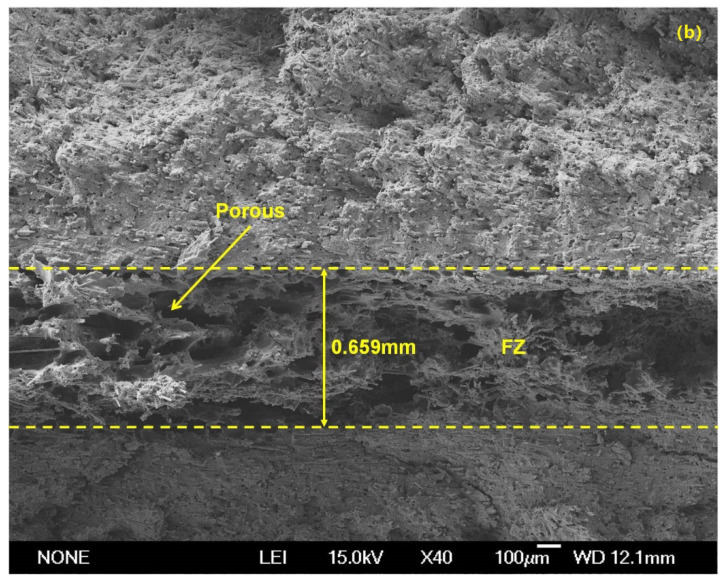
Microstructure of UW joints at weld time of 1.7 s (**a**) normal clamp (**b**) PPRC.

To further understand the effect of clamp on weld microstructure, the microstructure of UW joints made with a weld time of 1.7 s for the two clamps at were investigated, and the results were shown in Figure 12. Referring to Figure 12, both UW joints had a loose microstructure with pores in the fusion zone (FZ), however, the UW joints made with PPRC exhibited a thicker fusion zone and more pores than the normal clamp UW joints. Our previous study [14,19,28,31] indicated that the formation of pores in the fusion zone was mainly caused by the decomposition of matrix material during UW. Combined with the results from welding temperature at the faying interface, the transient temperature for UW joints made with PPRC during P2 phase was even higher than that for UW joints made with a normal clamp. Thus, the presence of loose microstructure with a great many pores in the fusion zone of UW joints made with PPRC was mainly caused by the high temperature at the faying interface.

## 4. Conclusions and Summary

In this study, extensive tests were conducted on ultrasonic welding of 4 mm thick composite with 30% weight fiber to investigate the effect of PPRC on weld quality. The conclusions are as follows:

1. For the given welding process various, PPRC UW joints had higher joint strength compared with the normal clamp UW joints. The strength of PPRC is 5.6 kN and 6.8 kN at weld time of 1.3 s and 2.1 s, respectively.
2. The applied force from the PPRC improved the contacting condition of upper and lower workpieces in the overlap region and consequently enhanced the energy dissipation at the faying interface.
3. The PPRC improved both the central weld nugget size and stable fusion region thickness, resulting in the formation of UW joints with good weld quality. When weld time is 1.3 s, the central weld nugget size and fusion region thickness are up to 286 mm^2^ and 0.63 mm, respectively.

Though the test results showed that the UW joints made with PPRC had a greater weld area, fusion zone thickness, and strength than the UW joints made with normal clamp under optimal process variables. However, it has some notable limitations in its practical application. Firstly, although the PPRC promoted the heat generation during the UW process, it likely produced obvious weld indentation of UW joints, which had an influence on the cosmetic appearance of the joints. Secondly, there are various contact conditions (i.e., off-angles between horn and workpieces or gaps between upper and lower workpieces, etc.) in actual welding process, the effects of PPRC on weld quality of joints with these loosen contact conditions need be further evaluated. Therefore, more studies, such as the optimization of weld parameters for it likely produced obvious weld identification clamp joints under various conditions (i.e., clamping force, off-angles, and gaps), and further analysis of heat generation behavior at the faying interface during the welding process will be required before applying it in the practical application.

## Figures and Tables

**Table 1:** Mechanical properties of molded 2.3-mm-thick Cf/Nylon 6 composite coupons.

	Tensile Strength (MPa)	Elastic Strength (MPa)	Poisson’s Ratio	Density (kg/m^3^)
Nylon6	74	2501	0.34	1130
C_f_/PA6	89.2	7532	0.34	1260

## Data Availability

The data presented in this study are available on request from the corresponding author.
